# Contextualizing willingness to participate: recommendations for engagement, recruitment & enrolment of Kenyan MSM in future HIV prevention trials

**DOI:** 10.1186/s12889-017-4395-4

**Published:** 2017-05-18

**Authors:** Monika Doshi, Lisa Avery, Ronnie P. Kaddu, Mary Gichuhi, Gloria Gakii, Elsabé du Plessis, Sumit Dutta, Shamshad Khan, Joshua Kimani, Robert R. Lorway

**Affiliations:** 1 The Centre for Global Public Health, University of Manitoba, Community Health Sciences, R070 Med Rehab Bldg, 771 McDermot Avenue, Winnipeg, R3E0T6 Canada; 2 Saath, 50 South Highland Street, West Hartford, CT 06119 USA; 3 Aga Khan Hospital, Vanga Road, P.O. Box 83013–80100, Mombasa, Kenya; 4 Partners for Health and Development in Africa, Geomaps Centre, 4th Floor Wing B, Matumbato Road, Upperhill, Nairobi, Kenya; 5 Department of Medical Microbiology, University of Nairobi, P.O. Box 30197–00100, Kenyatta National Hospital Campus, Nairobi, Kenya; 6 Dr. K.N. Modi University, INS-1, RIICO Industrial Area Ph-11, Newai, Dist. Tonk, Rajasthan, 304021 India; 7 Department of Communication, University of Texas at San Antonio, One UTSA Circle, San Antonio, TX 78249 USA; 8 Department of Medical Microbiology, University of Manitoba, Room 543-745 Bannatyne Avenue, Winnipeg, R3E0J9 Canada

**Keywords:** HIV vaccine, Vaccine acceptability, Willingness to participate, Clinical trials, Men who have sex with men (MSM), Kenya

## Abstract

**Background:**

The HIV epidemic among men who have sex with men (MSM) continues to expand globally. The addition of an efficacious, prophylactic vaccine to combination prevention offers immense hope, particularly in low- and middle- income countries which bear the greatest global impact. However, in these settings, there is a paucity of vaccine preparedness studies that specifically pertain to MSM. Our study is the first vaccine preparedness study among MSM and female sex workers (FSWs) in Kenya. In this paper, we explore willingness of Kenyan MSM to participate in HIV vaccine efficacy trials. In addition to individual and socio-cultural motivators and barriers that influence willingness to participate (WTP), we explore the associations or linkages that participants draw between their experiences with or knowledge of medical research both generally and within the context of HIV/AIDS, their perceptions of a future HIV vaccine and their willingness to participate in HIV vaccine trials.

**Methods:**

Using a social network-based approach, we employed snowball sampling to recruit MSM into the study from Kisumu, Mombasa, and Nairobi. A field team consisting of seven community researchers conducted in-depth interviews with a total of 70 study participants. A coding scheme for transcribed and translated data was developed and the data was then analysed thematically.

**Results:**

Most participants felt that an HIV vaccine would bring a number of benefits to self, as well as to MSM communities, including quelling personal fears related to HIV acquisition and reducing/eliminating stigma and discrimination shouldered by their community. Willingness to participate in HIV vaccine efficacy trials was highly motivated by various forms of altruism. Specific researcher responsibilities centred on safe-guarding the rights and well-being of participants were also found to govern WTP, as were reflections on the acceptability of a future preventive HIV vaccine.

**Conclusion:**

Strategies for engagement of communities and recruitment of trial volunteers for HIV vaccine efficacy trials should not only be grounded in and informed by investigations into individual and socio-cultural factors that impact WTP, but also by explorations of participants’ existing experiences with or knowledge of medical research as well as attitudes and acceptance towards a future HIV vaccine.

*Interviewer (community researcher and sex worker)*: What do you think that the people in charge of this kind of research [clinical trials/HIV prevention trials] need to do with the MSM community before they start the research?

*Participant (Mombasa, 20 years)*: They should explain to them what the research is about – not enrolling someone into research because he is an MSM. He [the potential study participant] keeps hearing there is a research [study] that is starting, that there is money – one thousand or two, three thousand – he will run for the money…because it is someone’s life you have to be sure of what is going on…. You run for the better option because research comes in every type and researchers are everywhere in town.

## Background

The HIV epidemic, which began amid a biomedical crisis, has been transformed over the past three decades with successive advances in treatment, care, and prevention. In the fight against one of the most intractable human epidemics, there are now various technologies and techniques available to public health scientists which can be classified as behavioural [1, 2], structural [3, 4] and biomedical interventions [5, 6], with PrEP (pre-exposure prophylaxis) being the most recent addition to combination prevention. Despite these developments, HIV infection rates remain high particularly in low- and middle- income countries with men who have sex with men (MSM) disproportionately affected compared to other groups at greater risk for HIV infection [7–9]. Although research among MSM has been limited especially in low- and middle- income countries, findings from past studies show that a range of factors impact their vulnerability to HIV. High biological risks of HIV transmission associated with anal intercourse coupled with individual risks, such as unprotected receptive anal intercourse, high number of lifetime partners, alcohol & drug use, and high frequency of male partners, have been well documented [10, 11]. In addition to biological and behavioural factors, human rights violations, homophobic policies, cultures of discrimination, stigma and violence, particularly in the African context, have also been found to negatively influence the health status of MSM including their vulnerability to HIV infection [12–15]. Although unlikely to be a panacea for prevention, the impending discovery and addition of an efficacious, prophylactic vaccine to combination prevention offers immense hope for strengthening the response to HIV/AIDS. As the newest promising HIV vaccine moves into the human testing stage [16] and with nearly three dozen other vaccines in some stage of human trials, there is a continual need for volunteer communities, that is groups of individuals who identify with specific communities (e.g., MSM, FSWs – female sex workers) and consent to participation in clinical trials, to test vaccine efficacy. Through an assessment of motivators and/or barriers that influence people’s willingness to participate (WTP), vaccine preparedness studies have informed strategies towards the engagement of communities and recruitment of trial volunteers [17–19]. However, in low- and middle- income countries, there is a paucity of vaccine preparedness studies that specifically pertain to MSM and other communities at greater risk for HIV exposure—that is, those that are the most likely to benefit from an efficacious vaccine.

The few studies focused on the willingness of MSM in low- and middle- income countries to participate in HIV vaccine trials have examined the rational perceptions of individuals by presenting research participants with trial attributes and characteristics. Side effects, vaccine-induced seropositivity, access to efficacious vaccine posttrial, distance to trial site, type of venue, and financial incentives were among the factors found to greatly impact WTP; see for example: [20, 21]. Additionally, systematic reviews of vaccine preparedness studies conducted by Dhalla and colleagues [18] found that both personal (i.e., monetary incentives, protection from HIV) and social benefits (i.e., altruism) were motivators of WTP. Few studies have contextualized the factors that influence decision-making. The study by Chakrapani and colleagues [22] is a notable exception, where spheres of influence around WTP included social-structural, community, and family. Factors such as family discord, partner rejection, fear of within group discrimination, disclosure of same-sex sexuality, gender non-conformity stigma, and institutionalized discrimination were found to be associated with WTP. Existing vaccine preparedness studies have been pragmatic in their explorations and integral in appraising individual and socio-cultural motivators and barriers that influence WTP.

Our study, Vaccine Acceptability among Stigmatized Populations (VASP), is the first vaccine preparedness study to be conducted among MSM and FSWs in Kenya. In this paper, we focus on the willingness of MSM to participate in HIV vaccine efficacy trials. As with past studies, we examine the individual and socio-cultural motivators and barriers that have often been investigated in other settings to inform strategies towards engagement of communities and recruitment of trial volunteers. Unlike past studies, we explore the associations or linkages that participants draw between their experiences with or knowledge of medical research both generally and within the context of HIV/AIDS, their perceptions of a future HIV vaccine and their willingness to participate in HIV vaccine trials. We argue that strategies for engagement of communities and recruitment of trial volunteers should not only be grounded in investigations around WTP in HIV vaccine efficacy trials but they should also be informed by the participants’ experiences/knowledge of medical research as well as their attitudes and acceptance towards a future HIV vaccine. Furthermore, we explore community engagement not just as a precursor to trial recruitment but in the context of sustainable partnerships that advance the interests and the role of MSM in HIV vaccine clinical trials and vaccine dissemination.

### Background of HIV-related medical research in Kenya

The distinctive history of medical research in Kenya, specifically in the context of HIV/AIDS, serves as the backdrop against which to further contextualize the participants’ responses in relation to their willingness to participate in HIV vaccine efficacy research. Among countries that are home to medical research, Kenya has had a long and complex legacy with respect to HIV research. This legacy has arguably shaped the ethos of the renowned Kenyan HIV vaccine research program that has emerged over the past decade, established by Kenyan scientists and their (mostly) Canadian and American collaborators.

Medical research in Kenya was once rooted domestically with the Ministry of Health at the helm. The late 1970s saw a paradigm shift as ‘parastatal’ research institutes, such as the Kenya Medical Research Institute (KEMRI), began to gradually draw control of medical research away from the government and local academic institutions through collaborations with transnational partners [23]. Among the transnational collaborations that emerged in the early 1980s, the University of Manitoba and the University of Nairobi formed a notable scientific partnership. The partnership began with the Majengo Observational Cohort Study (MOCS), a study focused on examining sexually transmitted infections (STIs) among FSWs in an industrial slum on the edge of Nairobi. Although credited with major scientific breakthroughs in HIV prevention during the first few years of the study [24, 25], the investigators have also faced international criticism over the potential exploitation of the study participants. Some critics have raised concerns over the investigators’ ethical priorities in allowing participants to continue in the study cohort for decades, questioning whether the women served as “sex slaves for science” and whether scientific progress and interest transcended the fundamental principles of research ethics [24]. Others have emphasized the vulnerability of the women as research participants [26], including the exclusion of representatives from sex worker communities during discussions on negotiations, decision-making, and benefit-sharing [27]; standard ethical issues that not only influence the conduct of research studies but also inform study participants’ willingness to participate. Furthermore, as noted in the case study by Bandewar and colleagues [28], the women in the study cohort themselves voiced disappointment that their participation hadn’t resulted in their liberation from sex work. In the absence of protocols related to medical research with human subjects specific to the African context, investigators struggled to be ‘ethical, sensitive and effective’ as they navigated issues related to autonomy of the participant, informed consent, capacities of the host country (Kenya), transparency, and establishing equal partnerships with their international counterparts [25]. As the medical science landscape in Kenya evolved, concerns over the ethical conduct of trials were often compounded by those over ownership and intellectual property rights between collaborating institutions [29]. The resulting unequal power relations between partners from the global north (donors/researchers) and those from the global south (recipients/participants) have come to characterise the contemporary landscape of medical research in Kenya.

The turn of the century ushered in an intensified focus on clinical trials in Kenya, reinforcing unequal transnational collaborations through ‘parastatals’, and further shifting power away from the national government toward European and North American academic institutions that often designed the trials. In the context of HIV prevention research, Kenyan communities have volunteered in trials related to PrEP [30], male circumcision [5], and HIV vaccine candidates [31–33]. The latter, influenced by a shift in HIV prevention priorities in the global north to HIV vaccine development, resulted in a multitude of transnational partnerships, including those among the University of Nairobi whose list of foreign collaborators expanded to include the International AIDS Vaccine Initiative (IAVI). Since 2001, the Kenya AIDS Vaccine Initiative (KAVI), in collaboration with its funding agency IAVI, has conducted phase 1 and phase 2A HIV vaccine trials among participants considered to be at low-risk behaviour for HIV and other sexually transmitted infections [34]; the exception being vaccine trials centred on investigating the immunologically protective mechanisms found in the Majengo sex workers. Similar to early medical research, engagement of communities in these vaccine trials has been limited to support trial enrolment with study participants offered medical care, among other incentives, for the duration of enrolment. Despite gains in knowledge and experience with medical research in low- and middle- income countries, things remained the same (e.g., unequal partnerships – between institutions, as well as between institutions and communities, minimal engagement of trial communities) until more recently. With broadening institutional interests, the University of Manitoba has prioritized expanding community engagement beyond study recruitment, with a commitment to equalizing community-institution partnerships and building capacities of community partners, especially in the context of scientific research. It is within this more equitable framework that our team launched this study.

## Methods

The VASP study was a multi-country research programme which included China, India, and Kenya. As a mixed-methods study, it was comprised of different interconnected research phases including a qualitative phase (i.e., primary and secondary historical research, ethnographic field notes and in-depth interviews) and a quantitative phase (i.e., quantitative survey). Although the study consisted of components that stood alone as either qualitative or quantitative studies, findings from each preceding phase were used to inform the next phase.

Our research programme focused on selected communities of MSM, FSWs, and frontline health service providers (FHSP) at 3 sub-sites within each study country, respectively. The Kenya study sites included Kisumu, Mombasa, and Nairobi. These three cities have historically been and continue to be important centres for HIV research. Cognizant of the historical precedence of medical research in Kenya and fully invested in a community based approach, we directly engaged members of MSM communities in our study. The Kenyan MSM research team consisted of 7 community researchers (CRs) who were selected from the respective MSM communities in Kisumu, Mombasa, and Nairobi. Frontline organizations working with MSM were engaged to help identify community researchers. Those selected as CRs were well-respected leaders in their communities, were experienced in sexual health research and/or programming, and played a central role in the design of data collection tools, collection of data by conducting qualitative interviews, as well as data analysis. As members of the sex worker community, the CRs were familiar with and sensitive to the lived experiences of the participants. In another context, Lorway et al. found that directly engaging MSM communities in research helps to tackle stigma and discrimination as key methodological obstacles [35].

Prior to data collection, the CRs participated in a training workshop led by members of the research team. Training covered qualitative research methods, basic interviewing skills, and research ethics. The workshop also focused on cultivating leadership in public health research and building capacity in HIV vaccine knowledge; both objectives were accomplished through tangible presentations and discussions that de-mystified clinical trials and HIV vaccine research. Data collection was standardized across countries, although limited modifications to study tools were made to accommodate some country-specific investigations.

### Study design, sampling and recruitment

As part of the larger study, this phase was comprised of semi-structured qualitative interviews. The interview guides were initially developed by the first and last authors, in partnership with the CRs, and were informed by the analysis of field notes from the ethnographic phase of the research programme. They were then pilot tested and finalized for implementation following consultation on wording/phrasing with the CRs post pilot testing. The interview guides were translated into Kiswahili. Interviews were conducted in the participant’s preferred language – either Kiswahili or English.

Utilizing a social network-based approach, we employed snowball sampling to recruit MSM into the study. During the first round of recruitment, the CRs, who were diverse in age, socio-economic status, education, and sex work typology, recruited individuals from their social networks. Participants from each subsequent round of recruitment referred individuals in their networks to the CRs. Recruitment continued until a sufficient and representative sample size was reached, as reflected in the social networks of the CRs (i.e., age, socio-economic status, education, and sex work typology). The study coordinators were responsible for ensuring that participants did not overlap between CRs. All participants were 18 years of age or older and were able to provide informed consent. A total of 70 MSM were interviewed from Kisumu (*N* = 20), Mombasa (*N* = 20), and Nairobi (*N* = 30). Participants were provided with an honorarium of 400 Kenyan Shilling (KES), approximately 4 USD, for their time and expenses related to their participation.

### Data collection and analysis

The CRs collected narrative information directly from the MSM through individual in-depth interviews (IDIs) using semi-structured interview guides containing mainly open-ended questions and some prompts. Interviews were framed in a mutually respectful, non-judgmental context. Participants were led through a series of discussions related to their general knowledge, cultural perceptions, and levels of trust in relation to existing vaccination programs, public health interventions, biomedical science, and traditional medicine. Discussions also centred on their experiences with and attitudes towards clinical trials, HIV prevention trials, and future vaccines. All interviews were audio recorded and held in locations that maximised participant confidentiality and safety. Prior to the interview and following informed consent, participants completed a brief survey which was used to collect demographic information such as age and education.

Interviews were transcribed verbatim with those conducted in Kiswahili translated into English. All transcripts were reviewed by the first author, who imported them into NVivo 9 [36] for analysis. Through broad-brush coding, we organized the data into topic areas/themes covered by the interview guide. Transcripts were analyzed thematically [37]. This process (led by the first author) involved initial open coding of data under each interview topic area, identifying the node structure by assigning concept-specific nomenclature and definition. The first and last authors independently carried out detailed coding through the use of the finalized coding scheme. The minor discrepancies that arose between coders were resolved through discussions with the second author.

## Results

### Profile of participants

A total of 70 MSM were interviewed. Nearly two-thirds (64.3%) were 18–25 years of age with a mean age of 24.4 years. All participants reported having exposure to formal education with nearly one half (48.6%) starting secondary education and a majority (94.1%) among them having completed it. Most participants (70%) reported being single or never married. Nearly one-half (44.1%) reported that their main source of income was from sex work. Among those who reported sex work as the primary source of income, nearly one-third (30%) reported a secondary source of income to supplement earnings from sex work. Of those participants who reported a primary income source other than sex work, more than one-half (55.3%) reported relying on sex work as a supplementary/secondary source of income with the remaining participants either reporting another source of secondary income (7.9%) or no supplemental/secondary income (36.8%). Among participants who reported practicing sex work, over one-half (56.9%) had done so for 1–5 years and over one-third (36.2%) for 6–10 years. The household average monthly income varied among participants, with nearly one-half (44.8%) reporting less than 10,000 KES (~100USD).

### Experiences with clinical trials/medical research

The vast majority of study participants had either a limited technical understanding or no technical understanding of clinical trials/medical research, despite the fact that Kenya has been a vibrant centre for scientific studies involving human subjects since the 1980s. Irrespective of the study’s geo-location, whether Kisumu, Mombasa, or Nairobi, participants were unable to identify and convey their comprehension of the phases associated with clinical research. However, participants demonstrated their understanding through a historical lens, through the experiences of their friends/relatives or through their own personal experiences. Their knowledge was grounded in the context of national non-governmental institutions, recognizing the central role they played in scientific studies involving human beings. For a number of participants, institutions such as KEMRI and KAVI, as well as their international benefactors such as the United States Centers for Disease Control and Prevention (CDC) and IAVI were synonymous with clinical trials/medical research. They were identified and regarded as institutions exclusively conducting “research on human beings”*.*

A small number of participants reported ever having been enrolled in human trials. Those who did participate did so in studies centred on HIV prevention, such as PrEP or male circumcision. Although they generally reported having positive experiences – void of discrimination, grounded in confidentiality, transparency, and access to information as well as ample compensation, both monetary and through the provision of health services and other subsidies – their engagement and role usually never extended beyond that of study subjects.

*During that research [circumcision trial], there was medical cover for those who did it for five years. So anything that happened to you, they were responsible and we were taken to big hospitals when we had anything. You were even given big money in case you went for the visits. It was like you were being given like 2K per visit and the visit was after every one and half months. [Kisumu, 19 years]*

One participant did report serving in an advisory capacity, however, his role primarily centered on information dissemination and communication while interfacing between the study population, namely an MSM community, and the research team.

*I have participated in research work which is done by KAVI and IAVI. They are trying to develop AIDS vaccines. I have worked in the community advisory council where we collect information from the community or you advise the community regarding what is entailed in regard to that particular research and trying to disseminate information correctly…. Personally, I have been a part in terms of giving out information, advising the researcher, and being a link actually to the community that is being researched, that being the MSM. [Nairobi, 32 years]*

Once the interviewer defined the term ‘clinical trials/medical research’, the vast majority of the study participants raised concerns when asked whether such research caused more benefit or harm to the MSM community. The participants either shared their personal experiences or that of others that they knew. Some participants voiced apprehension around unwanted side effects related to clinical trials/medical research; however, most participants’ disquietude centred on the (mis)use of MSM as test subjects/guinea pigs.

*Ok, you know most of the research coming to Kenya starts with MSM. Those are the ones that are tested on first so if there are side effects, those will be the first victims. [Nairobi, 20 years]*

*I think it should be tested universal not direct to one community alone. It should be universal where anybody who feels like he wants to be tried should go and not be directed to a certain community…I have seen they are coming too much to the MSM community. So, I would prefer a little change so that it would be universal. [Nairobi, 24 years]*

*If the AIDS vaccine comes out then it is for the general public, but at the same time what is there for the MSMs? I mean they are just going to be used as guinea pigs or lab rats….What is there for the MSMs, are they going to be pioneers of this thing or are they just going to be those people we used along the way to get what we wanted…. How well is their health taken care of there after? You start with someone, for AIDS vaccine, you need someone who is both positive and negative and at some point you need more negatives than positives. So when you acquire HIV, what happens to you during that study? [Nairobi, 22 years]*

The benefits of clinical trials/medical research, outside of access to free and non-stigmatizing healthcare and treatment which was experienced to be otherwise elusive among participants, were largely seen to profit the general population in terms of finding medicines, gaining scientific insights, and finding cures to various high burden diseases. Unlike the relative low literacy around clinical trials/medical research generally, participants’ knowledge and awareness around HIV vaccine research was comparatively high.

### Knowledge of and attitudes towards HIV vaccine research

The majority of MSM interviewed were aware that an HIV vaccine did not exist and some even cited antiretroviral (ARV) drugs as the only treatment currently available to combat HIV. Participants’ knowledge around the search for an HIV vaccine was demonstrated by their recognition of on-going efforts mostly met with failure and set-backs. They specifically recalled global and local attempts towards the discovery of an HIV vaccine and identified KAVI and KEMRI, often in partnership with foreign entities, as the key investigators in Kenya.

*Yeah, I read it in the press…I think the Kenyan doctors and the American doctors are supposed to be developing the HIV vaccine…an NGO called KAVI in Nairobi was engaged in that project. [Kisumu, 22 years]*

*I know of the KEMRI CDC research on the vaccine for HIV and AIDS. [Kisumu, 29 years]*

One participant’s recollection of local HIV vaccine trials, coupled with the fact that experiences with clinical trials/medical research among the MSM interviewed primarily involved HIV prevention studies, underscores the pivotal role that key populations continue to play as study participants, including in HIV vaccine research.

*There is another one [study] conducted in Majengo, in Nairobi, with sex workers…the intention was to find out if these women were given this vaccine then they cannot contract HIV. I also heard about another [clinical trial] sometime back that was conducted in Nairobi by the MSM community. [Kisumu, 28 years]*

#### Perceived benefits of a future HIV vaccine

Most participants felt that an HIV vaccine would bring a number of benefits to self, as well as to MSM communities. For many participants, especially among those who relied on sex work for their livelihood, a vaccine would alleviate the fear of contracting HIV, enable engagement in sexual activity without ‘falling sick’, and facilitate income generation by securing worry-free avenues to ‘making money’.

*I think it will be beneficial by the kind of work we are doing of sex work. You need to protect yourself and you don’t know the needs of clients. You don’t know what will happen so as a sex worker, I know I am at risk because of exposure. It will prevent me from acquiring the HIV disease. [Kisumu, 22 years]*

*It will be good because there will be no falling sick and then we will do in our own pleasures…. Even if you want [to practice sex work] seven days, seven times, three times, you know our jobs now there will be no worry. [Mombasa, 25 years]*

An HIV vaccine, according to participants, would also facilitate engagement in sexual activities without always requiring, planning for, or negotiating the use of condoms. Some MSM acknowledged that there was a strong preference within the community to not use condoms. The prospect of an HIV vaccine was seen to support this preference. Others also reflected on past situations when condom use was shadowed by ineffective decision-making due to alcohol intoxication – a scenario which was acknowledged to increase HIV-acquisition risk but a moot one in the presence of a vaccine, according to participants.

*It will benefit MSM community because the majority does not like to use condoms and it would be good; it would prevent them from HIV and spreading HIV. [Nairobi, 26 years]*

*It will benefit many of us…on my side…because sometimes I’m drunk I go out and meet people and they tell me they do not use condom…or… I’m drunk, I don’t know myself and I have already come to the bed with someone. Even I don’t know what he will do to me, if he will do me with a condom or if he will do me without a condom. Now the [HIV] vaccine…will be beneficial to me and the whole community. [Mombasa, 23 years]*

In addition to quelling personal fears related to HIV acquisition, participants also felt that a vaccine would benefit the MSM community by reducing HIV incidence and prevalence, by preventing deaths, and by promoting longevity. Many participants recognized the burden HIV imposed upon their community. They viewed the future vaccine as a means to address and eventually eliminate vulnerability to HIV – a means to address morbidity and mortality within their community thereby promoting life expectancy.

*I think it will prevent them [MSM] from getting infected with HIV which has been like one of the biggest problem in the community. [Nairobi, 27 years]*

*It would be very beneficial to the MSM community. This then would reduce the infection rate among MSM or better yet eradicate the infection rate in the MSM…the HIV [virus] will be rendered invalid. [Kisumu, 22 years]*

*It would be a great benefit because it would lower the deaths in the MSM community. [Nairobi, 22 years]*

In addition, some participants felt that a vaccine would help reduce or altogether eliminate the often devastating stigma and discrimination shouldered by their community, specifically in the context of being viewed as the community that ‘spreads’ HIV/AIDS.

*They [MSM] will be comfortable…people will stop discriminating…you MSMs are the ones with HIV and you are the ones spreading it…those stigma they will not get again. [Mombasa, 19 years]*

#### Perceived detriments of a future HIV vaccine

Alongside the benefits, there were pauses for caution among the participants. An HIV vaccine was also seen to potentially have negative consequences on their community both during the actual trial or testing period, as well as once it was ready for uptake. Participants questioned both the potential efficaciousness and effectiveness of an HIV vaccine and the associated potential threat to the MSM community.

*Vaccine might not work on all strains you know, and it would be a big blow to the MSM community. [Nairobi, 26 years]*

*When the vaccine is introduced, they [MSM] would not take much serious…they will have something like unprotected sex which might cause more harm…some will take the vaccine to be 100%…it kind of never worked and actually can cause more harm to the MSMs. [Kisumu, 20 years]*

While a few participants pondered the gravity of the consequences on communities at high risk for HIV if a vaccine wasn’t 100% effective and didn’t address multiple strains, the vast majority voiced concern over its potential negative influence on sexual risk taking behaviours. Participants believed that the benefits of an HIV vaccine would be undermined through risk compensation (i.e., members of their community taking advantage of a vaccine’s protection to engage in more risky behaviour than they would otherwise) and further exposure to sexually transmitted infections not targeted by the vaccine. Participants expressed concerns over an increase in unprotected sex, in high-risk sexual activity and in the number of sexual partners among members of the MSM community.

*It will be easy for us to contract those STIs…. We will be thinking to have sex more often. These condoms, there will be none…. You don’t know someone may have this disease. He comes and infects you. You are not worried. You don’t go to the hospital. Right now, this HIV makes us go to hospital every time. But there is nothing to harm me, I do in my pleasures. When I see the hospital there, I pass it. You see others will not use condoms…they will be more exposed. [Mombasa, 23 years]*

Participants also felt that an HIV vaccine could negatively impact their community by promoting a false sense of protection.

*…now the problem is of course people will still continue to engage in reckless sexual behaviors and the fact that using protective measures prevents or protects against other infections so the fact that you are using HIV vaccine, that doesn’t vaccinate you against gonorrhea, syphilis and others. So for me the fact that now people will feel safe…the cases of other STIs would likely to increase because if you assure this person that you will not get HIV then the rest they can risk. [Nairobi, 27 years]*

*…people will be sleeping with HIV infected people because at the end of the day, I have vaccine in me. [Nairobi, 26 years]*

### Willingness to participate in future HIV vaccine trials

An overwhelming number of participants expressed willingness to participate in future HIV vaccine trials, though not without reflection on ways the vaccine would positively and negatively impact their community, the MSM community. Despite the potential negative ramifications of a future HIV vaccine, the vast majority felt that their role as study participants in HIV vaccine research would contribute to downstream advantages, as opposed to disadvantages, for the MSM community. Furthermore, they not only viewed their participation in HIV vaccine efficacy trials as having a positive impact on the larger community, but they also felt that they would benefit on an individual level. The desire to help their community, a community disproportionately affected by HIV, was the foremost reason for willingness to participate in future HIV vaccine trials.

*I would like to participate…it is something very important. I will not benefit myself, but there is a generation which we are praying will not be affected like us…. I would want very much it be like a legacy, there are certain people who contributed a lot to finding a HIV vaccine. [Mombasa, 25 years]*

For some participants, their willingness to enroll in HIV vaccine clinical trials was also inherently linked to a nationalistic and altruistic sense of duty.

*Actually, I would participate in order to support my MSM community…and the nation at large; you know when you participate, you are patriotic to your country. [Nairobi, 24 years]*

The participants’ altruistic tendencies towards willingness to enroll in future HIV vaccine trials often extended beyond their desire to help the immediate community of family, friends, and fellow MSM to include the larger society and at times the nation and even the world*.*

*Because you know…the prevalence of HIV in MSM is high and getting that vaccine would really mean a lot not only to the MSM community but to the community and public. [Nairobi, 26 years]*

Moreover, in addition to the perceived positive outcomes their enrolment in HIV vaccine research would have on the MSM and larger community, participants felt that they would also benefit personally. Through their enrolment, they felt that they would be able to protect their own health, gain access to pertinent information, and be involved at the forefront of integral research.

*I would like to participate because well as an MSM, being in the MSM community, I’d like to prevent myself from getting infected…being a sex worker and sleeping with multiple partners…I have to be part of that so that I can help myself. [Kisumu, 20 years]*

*I would like to be a role model and one of the pioneers. [Kisumu, 26 years]*

Whether for self, for the MSM community, for the nation, or for the global community, the urgency of finding an HIV vaccine was consistently expressed among participants. Most recognized the critical need for an HIV vaccine and hence vaccine research; however, their willingness to participate in the trials was also found to be governed by non-negotiable principles inextricably tied to researchers’ responsibilities before, during, and after a clinical trial. For study participants, the willingness to volunteer for HIV vaccine efficacy trials hinged on whether the researchers would honour their responsibilities.

Participants emphasized that the utmost priority of any medical research should be given to the person – the so-called “study subject”. The health and well-being of study participants superseded all else*.*

*As far as I am concerned, they [researchers/research staff] should first of all care mostly for the health of MSM rather than just the outcome of the study. I think that should be a key priority in all aspects…that these people [study participants] do not in any circumstance acquire HIV because of the research. [Nairobi, 22 years]*

Safeguards such as confidentiality and privacy were also identified to be critical towards ensuring the well-being of study participants*.*

*They [research staff] should put their [study participants] more personal details in privacy…they should consider privacy…not disclose their information like to the media. [Nairobi, 22 years]*

The rights of individuals, specifically those belonging to marginalized communities, were seen to require special attention*.*

*The interaction should be in a friendly way…the medical practitioner or the researchers should be friendly. The society at large view the MSM community to be so marginalized and to be so ungodly so they withdraw their service from the point of perspective of people being bad…so they are going to handle them in a negative way. I feel maybe the MSM community is special and it should be handled with care. [Kisumu, 22 years]*

Some participants recalled past instances where the health and well-being of study participants were felt to be compromised.

*I will focus on KAVI…and MSWs…a guy went there, signed the form…was given the placebo or the test but it affected him…because of the release form, they [research staff] never bothered…. [Nairobi, 26 years]*

In addition, participants stressed the importance of transparency and disclosure, identifying them as key ethical responsibilities of any researcher. Furthermore, participants considered transparency and disclosure as non-negotiable with respect to their willingness to participate in HIV vaccine efficacy trials*.*

*…before you even test me, give me the information, the possible side effects and everything. [Nairobi, 26 years]*

A vast majority of the participants felt that all information related to the research study needed to be accurate, clear, concise, and communicated in an open and timely manner*.*

*Inform them [MSM], give them pros and cons, give them the possibilities and likelihoods of actually acquiring HIV, clear out the myths. [Nairobi, 22 years]*

*They [researchers/research staff] need to create that rapport; they need to give feedback constantly whether it is negative or positive…. They should not spoil by just cutting the link all of a sudden…it creates an impression where people feel they were just used; they are guinea pigs. [Nairobi, 32 years]*

In addition to maintaining transparency and open communication, some participants stressed the importance of establishing equal partnerships between trial communities and the researchers, particularly underscoring the value of involving members of the trial communities from the beginning*.*

*They [researchers/research staff] need to sensitize them [MSM] and get their consent and in fact actively involve them in drafting of the issues of the research. They [MSM] also need to take part actively so far as drafting and every decision [that] is made. [Kisumu, 23 years]*

Moreover, capacity building of community partners was seen as integral component of establishing partnerships.

*…try getting them involved not only at the levels of just them coming and maybe giving you samples.... [Nairobi, 27 years]*

Lastly, participants expected researchers to maintain investment in communities even after completion of a study. They viewed this to be critical not only with respect to dissemination of research outcomes but also in terms of protecting the health of study participants long-term.

*They [researchers/research staff] should come up with an exit plan, a clear exit plan that within this time we will be offering you medical care up to this period so that to counter side effects of the research that may arise after the research. [Kisumu, 23 years]*

For some participants; however, enrolment in HIV vaccine research could never be a feasible proposition either due to unwanted side effects or for the reason that they did not want to serve as ‘guinea pigs’.

*No, I am not ready…. This is a new thing…okay, yes, HIV virus must be tested through a human being…but for me, no. [Nairobi, 24 years]*

## Discussion

This qualitative study is the first vaccine preparedness study among MSM in Kenya, a strongly researched country particularly in the context of HIV/AIDS. The legacy of HIV/AIDS related medical research in Kenya can be characterised by ‘parastatal’ research institutes forging transnational collaborations and placing national research agendas in the hands of their wealthier Canadian, American, and other foreign partners. The resulting inequality in power between donors and recipients (local ‘parastatal’ research institutes and their community-based and non-governmental (CBO/NGO) organizational partners) not only vastly characterises the contemporary landscape of medical research in Kenya but also broadly defines the inequitable nature of relationships between researchers and study participants. In our study, participants reported experiencing limited engagement, one which usually never extended beyond that of a study subject, in clinical trials/medical research thereby prompting questions around fairness and equity [38], as well as revitalizing decades old unresolved issues related to decision-making and benefit sharing [27]. Furthermore, participants’ current concerns of being ‘guinea pigs’ echo those in the past raised by critics who questioned researchers’ ethics in allowing vulnerable populations to continue in studies long-term [24], as well as those who have challenged acceptance of off-shored HIV prevention research (by resource-rich nations) that does not directly benefit national health priorities and/or the study population/s [39].

The participants recognized that ‘parastatals’, not the government, play a central role in HIV prevention research in Kenya. The vast majority of the participants who had ever enrolled in clinical trials/medical research reported having done so in HIV prevention studies (e.g., male circumcision, PrEP), underscoring the continuance of a historical precedence for testing HIV prevention technologies among key populations not only in Kenya, but also globally. Our study participants’ knowledge and literacy around HIV vaccine trials, informed by the ubiquitous presence of ‘parastatals’, was grounded in the urgency of finding a cure for a disease that affected their community disproportionately. Willingness to participate in HIV vaccine efficacy trials was highly motivated by what other researchers have characterised as various forms of altruism – including the desire to help the MSM community, family, friends, the nation, and the world; see for example: [18–21]. Protection from HIV was cited as a personal motivator for willingness to participate in HIV vaccine efficacy trials, a finding corroborated by Dhalla et al., [18] and Chakrapani et al., [22]. Specific researcher responsibilities centred on safe-guarding the rights and well-being of participants were also found to govern WTP among the MSM in our study. Participants also stressed the importance of transparency and disclosure during the research process citing personal instances or historical examples where violations in either led to an infringement of human rights. Furthermore, the participants believed that both transparency and disclosure could be maintained through clear, concise and timely communication. Another notable finding linked to WTP, grounded in breaking historical precedence and setting new norms, was the importance attributed by the participants in establishing equal partnerships between trial communities and researchers. In addition, towards the goal of level-setting power inequities, participants strongly felt that trial communities should be engaged in the research process from the beginning and that the engagement should be long-term, with researchers building capacity of the community throughout the engagement.

Among the participants in our study, willingness to participate in vaccine efficacy trials was also informed by reflections on the acceptability of a future preventive HIV vaccine. Perceived benefits to MSM communities, which outweighed the perceived detriments, included protection from HIV, alleviation of planning around condom use, release of HIV burden shouldered by the MSM community, and elimination of HIV/AIDS related stigma and discrimination encountered by the community. The perceived detriments of a future HIV vaccine, as also reflected in the findings from other studies, see for example: [40–42], included consequences related to partial efficacy and risk compensation. In addition to supporting strategies towards dissemination of a future HIV vaccine, these finding also illuminate the influence of a locally grounded cost/benefit analysis on WTP.

As a qualitative study with a relatively small sample size, generalizations certainly cannot be made beyond the participant group. Moreover, our findings may not be reflective of the views of MSM who are consistently more difficult to reach and therefore often underrepresented, if at all represented, in research studies such as ours. In addition, given the nature of the methodology used to ascertain information, namely through interviewers, there is a likelihood that participants may be inclined to give socially desirable responses. However, given that the subject of the study revolved around a “hypothetical future vaccine”, rather than personal information of a sensitive nature pertaining to sexual behaviour, we believe that the introduction of the related bias is minimal.

## Conclusions

Findings from our study uncover multiple influences on willingness of Kenyan MSM to participate in HIV vaccine efficacy trials. In addition to the individual and socio-cultural motivators and barriers, both perceived benefits of a future HIV vaccine and experience with/knowledge of medical research were found to influence their willingness to participate. Therefore, strategies towards community engagement and recruitment of trial volunteers should be informed by analysis and understanding of all three influences (i.e., individual/socio-cultural motivators/barriers, future vaccine acceptability, and experience with/knowledge of medical research) individually and in relation to one another. The legacy of medical research in Kenya implores a different and more equitable engagement of future HIV vaccine efficacy trial communities; an engagement which is more likely to succeed when commenced well before the design phase of the clinical research. Trial communities should be actively and equitably involved throughout the entire clinical research process. Investing time to understand the culturally specific histories of medical research will result in better design, implementation, dissemination, and uptake of the research as it will ensure that previous inequities, mistrust, and neglect are not disregarded nor repeated. This knowledge should be seen as a crucial sphere of influence that impacts future trial volunteers, the researcher, and future HIV vaccine efficacy research, intentionally or unintentionally, depending on the past history of medical research – whether it was experienced and/or perceived positively or negatively. Taking the time to understand the local context and to engage the community in discussion and planning around past issues would create a participatory research collaboration based on mutual trust, respect, and understanding; one that would support and promote ethically grounded, acceptable and sustainable strategies for dissemination and uptake of a future HIV vaccine. Furthermore, an equal and fair partnership would arguably promote an “active demand”, as opposed to a “passive acceptance” [43] of a future HIV vaccine over time by addressing and planning for elements that influence acceptance and demand such as supply-service factors (e.g., delivery infrastructure), social factors (e.g., agency of community members) and cultural factors (e.g., perceptions of vaccinations, perceptions of vulnerability).

## Abbreviations

AIDS: Acquired immune deficiency syndrome
ARV: Antiretroviral
CBO: Community-based organisation
CDC: Centers for Disease Control and Prevention
CRs: Community researchers
FHSP: Frontline health service providers
FSWs: Female sex workers
HIV: Human immunodeficiency virus
IAVI: International AIDS Vaccine Initiative
IDI: In-depth interviews
KAVI: Kenya AIDS Vaccine Initiative
KEMRI: Kenya Medical Research Institute
KES: Kenyan Shilling
MOCS: Majengo Observational Cohort Study
MSM: Men who have sex with men
NGO: Non-governmental organisation
PrEP: Pre-exposure prophylaxis
STIs: Sexually transmitted infections
VASP: Vaccine Acceptability among Stigmatized Populations
WTP: Willingness to participate
